# A Complicated Case of COVID-19-Induced Inferolateral Wall Myocardial Infarction Successfully Treated With Streptokinase

**DOI:** 10.7759/cureus.20348

**Published:** 2021-12-11

**Authors:** Taimoor Hussain, John Joyce, Salma Habib, Sohaib Tousif, Sunny Ratnani, Sneha Puvvada

**Affiliations:** 1 Neurology, Bolan Medical College, Quetta, PAK; 2 Medicine, MS Ramaiah Medical College, Bangalore, IND; 3 Medicine and Surgery, Institute of Applied Health Sciences, Chittagong, BGD; 4 Medicine, Ziauddin Medical University, Karachi, PAK; 5 Medicine, Saint James School of Medicine, The Quarter, AIA; 6 Medicine and Surgery, MS Ramaiah Medical College, Bangalore, IND

**Keywords:** covid-19-induced myocardial infarction, pakistan, therapeutic challenges of covid-19, covid-19, streptokinase for covid-19 mi

## Abstract

The treatment of myocardial infarction (MI) in coronavirus disease 2019 (COVID-19)-positive patients is both controversial and challenging, particularly in a healthcare setup unable to fulfill COVID-19 protocols. In this report, we describe a case of a COVID-19-positive patient admitted with COVID-19 pneumonia treated symptomatically with a non-rebreathing mask, dexamethasone, remdesivir, and low-molecular-weight heparin (LMWH). On day two of the hospital stay, the patient developed inferolateral wall myocardial infarction (MI) without hemodynamic instability. He was treated successfully with thrombolytic (streptokinase) with no severe complications. However, his hospital stay was further complicated by decreasing oxygen saturation and rising inflammatory markers including procalcitonin and IL-6, suggesting superimposed bacterial infection. Thereafter, he was placed on BiPAP oxygen, and aggressive antibiotic therapy including tigecycline along with clindamycin and moxifloxacin was initiated. He showed gradual daily improvements and was discharged after a prolonged hospital stay. To decrease the exposure and spread of COVID-19 infection among the healthcare workers, when there is a deficiency in medical staff, and no negative-pressure catheterization laboratory, thrombolytic can be used for treatment in low-risk, hemodynamically stable MI during this pandemic. However, this needs further research.

## Introduction

A number of complications of coronavirus disease 2019 (COVID-19) SARS-CoV-2 have been reported, including cardiac complications. According to a meta-analysis, “the most common complications were acute respiratory distress syndrome with 33.15%, while arrhythmia, acute cardiac injury, heart failure, and acute kidney injury were reported from 9% to 16%” [1]. According to a study, “cardiovascular complications also include myocarditis, acute myocardial infarction (MI), and venous thromboembolic events” [2]. In another large study of COVID-19 patients, “the fatality rate in those with coronary heart disease was 10.5% higher than the overall mortality rate of 2.3%” [3]. In addition, the social, economic, and psychological distress of the pandemic itself has resulted in stress-induced cardiomyopathy [4]. The treatment of COVID-19-induced MI is also a challenge given the ongoing pandemic and deficiency of medical staff, negative-pressure catheterization laboratory, personal protective equipment, and regular decontamination facility. We report a case of COVID-19 pneumonia in a 38-year-old diabetic and hypertensive male. He developed myocardial infarction subsequently on the second day of admission and was treated successfully by thrombolytic (streptokinase) without any severe complications. His hospital stay was further complicated by presumed superimposed bacterial infection and rising inflammatory markers, decreasing oxygen saturation. He was managed accordingly by an interdisciplinary team, and after a long 35-day hospital stay, he was discharged.

## Case presentation

A 38-year-old diabetic and hypertensive male came with complaints of shortness of breath associated with fever and cough for seven days. He was shifted to a tertiary care hospital from another center. He was suspected of bacterial and COVID-19 pneumonia. He was treated symptomatically with intravenous ceftriaxone, oxygen by simple mask, nebulized with ipratropium and beclomethasone, antihypertensives, and diabetes medications. Given the ongoing pandemic, a nasopharyngeal swab was taken, and he was tested for COVID-19 with reverse transcription-polymerase chain reaction (RT-PCR). His test was positive for COVID-19. He was thus shifted to the COVID-19 unit.

**Figure 1:**
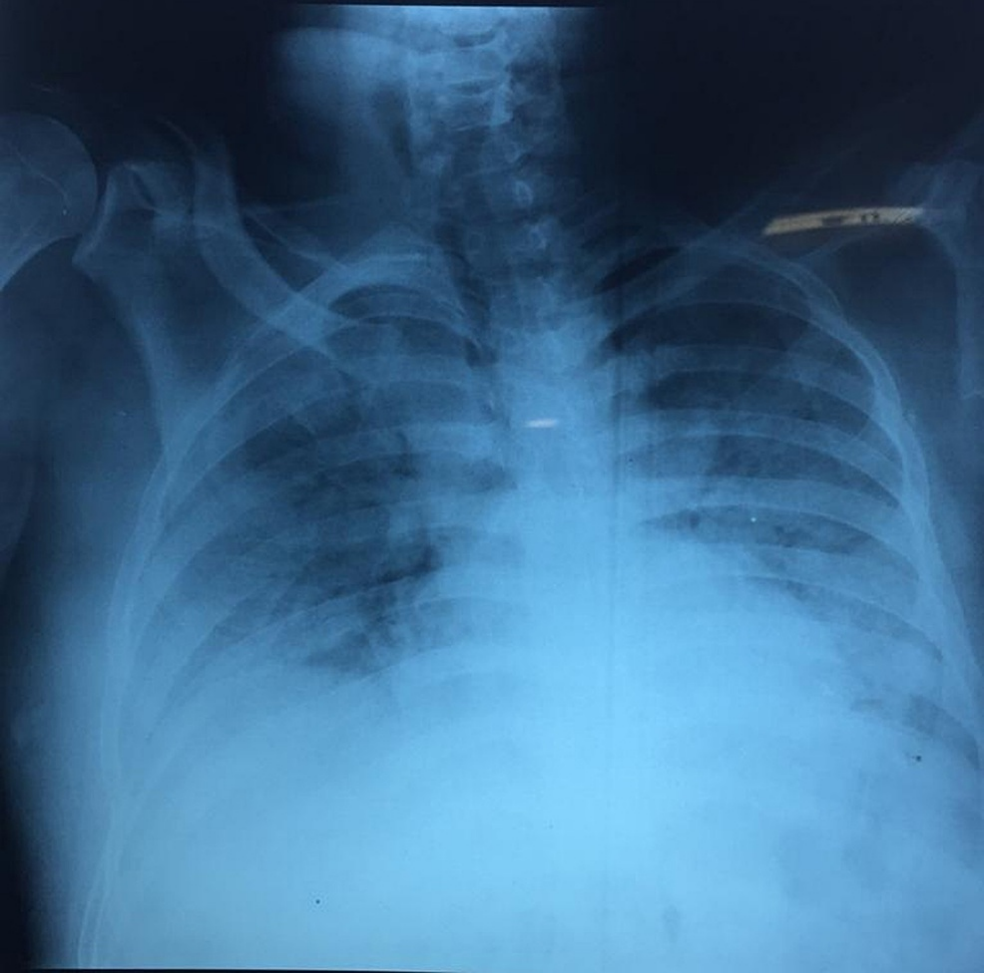
Chest X-ray showing multiple opacities

His initial laboratory tests were significant for leukocytosis; he has a WBC count of 20,900/µL, serum D-dimer levels of 305 ng/mL (normal range: <500 ng/mL), CRP of 73.09 mg/mL (reference range: 5 mg/L), and ferritin of 2000 ng/mL (reference range: 30-400 ng/mL). PT and aPTT, arterial blood gas analysis, and renal function tests were normal. He was vitally stable, except for oxygen saturation of 82%. The patient’s chest X-ray showed multifocal alveolar opacities, and he was diagnosed with multifocal pneumonia from COVID-19 (Figure 1). He was treated with oxygen using a non-breathing mask (NRM), intravenous dexamethasone, and remdesivir. Azithromycin was added to the treatment. He was also given S/C enoxaparin 40 mg once a day, vitamin C, and multivitamins.

**Figure 2:**
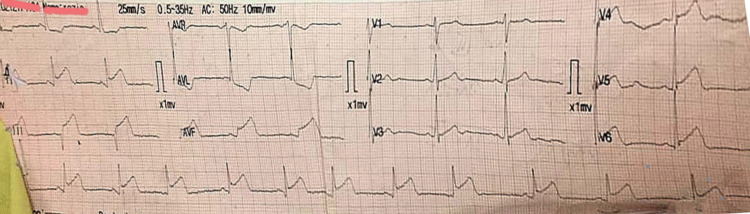
ECG showing ST-segment elevation in leads II, III, and AVf

**Figure 3:**
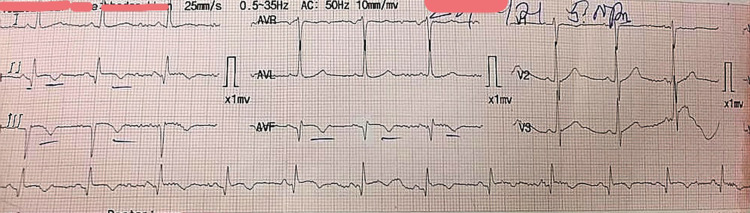
ECG after administration of streptokinase showing the resolution of ST-segment elevations

Two days after admission, he complained of chest pain and increasing shortness of breath. Electrocardiogram (ECG) was performed, which showed ST-segment elevations II, III, AVf, V5, V6, and T-wave inversions (Figure 2). Cardiac troponin I (cTnI) was 0.7 ng/mL (reference range: <0.6 ng/mL). He was diagnosed with inferolateral wall myocardial infarction for which he was given dual antiplatelet therapy (DAPT), low-molecular-weight heparin (LMWH), and statin as per the acute coronary syndrome protocol. He was then given fibrinolytic therapy with 1.5 million unit streptokinase over 45 minutes with continuous monitoring of vitals. Chest pain resolved but caused hypotension for which he was given intravenous normal saline. ECG was repeated after 90 minutes, which showed the resolution of ST-segment elevations (Figure 3).

Thereafter, he was started on dual antiplatelet therapy along with lipid-lowering drugs. His antihypertensive and diabetes medications were continued. Brain natriuretic peptide (BNP) was tested for risk stratification in patients with acute coronary syndrome. His BNP level was 64.5 pg/mL (normal range: <125 pg/mL). On the second day post-MI, his oxygen saturation dropped despite being on NRM oxygen therapy. ECG and relevant baseline investigations were repeated and found normal. However, inflammatory markers showed a rising trend, including IL-6 of 150.6 pg/mL (reference range: <7 pg/mL), serum procalcitonin of 0.67 ng/mL (reference range: <0.10 ng/mL), serum D-dimer levels of 2291 ng/mL (normal range: <500 ng/mL), CRP of 524.5 mg/L (reference range: <5 mg/L), and ferritin of 2000 ng/mL (reference range: 30-400 ng/mL). Therefore, a bacterial superinfection was suspected, and aggressive antibiotic therapy including tigecycline along with clindamycin and moxifloxacin was initiated. He was placed on BiPAP (20/10). He showed gradual improvement on a daily basis. His inflammatory markers showed a lowering trend. Chest X-ray showed resolution of opacities. He was shifted from BIPAP to simple mask oxygen. Finally, after 35 days of prolonged hospitalization, he was discharged. Currently, on follow-up visits, the patient was asymptomatic, had no complications from the dual antiplatelet therapy, and was advised coronary angiogram.

## Discussion

As the pandemic continues, a number of COVID-19 complications are reported, and therapeutic concepts have evolved. Acute myocardial injury in COVID-19 is also being reported increasingly for which different mechanisms are proposed. According to a case series of 5700 patients, a high number of COVID-19 patients have cardiovascular risk factors including hypertension (56.6%), obesity (41.7%), and diabetes (33.8%) [5]. The proposed mechanism of COVID-19-induced MI is summarized as follows. The hypoxic state and systemic inflammatory response due to the cytokine storm cause endothelial damage and cardiac ischemia; activated macrophages secrete collagenases that degrade collagen, a major constituent of the fibrous cap on atherosclerotic plaques, destabilizing the atherosclerotic plaque, and tissue factor that is a potent pro-coagulant leads to a state of hypercoagulability and thromboembolic events [6-9]. Bioinformatics studies suggested that some binding proteins and viral envelope glycoproteins may bind to both porphyrin and the β-chain of hemoglobin, decreasing available serum hemoglobin, consequently leading to hypoxemia and type 2 AMI, and hypoxemia, with pulmonary and cardiac consequences [6,10]. In addition, angiotensin-converting enzyme 2 (ACE-2) signaling pathways are damaged from direct damage to ACE receptors on myocytes [11].

According to a study “in 201 COVID-19 patients with myocardial injury, elevation in CKMB and troponin I levels was found in all of them; 43.7% presented with new electrocardiography (ECG) changes, 36.3% had ST depression, and 18.7% of the patients had abnormal echocardiography findings, and right ventricular dilatation and dysfunction were commonly seen in the critical group patients” [12]. cTnI, cardiac troponin (cTnT), and BNP have shown remarkable potential in predicting COVID-19 outcomes and deteriorating health. Those with higher cTnI levels have statistically significantly higher mortality as compared to others with normal levels [13]. Therefore, detecting elevated serum cTnT or cTnI levels on admission as a routine procedure has immense value in reducing mortality.

The treatment of COVID-19-induced myocardial infarction is controversial. In patients diagnosed with an ST-elevation myocardial infarction (STEMI) and COVID-19, the American College of Cardiology (ACC) recommends “that percutaneous coronary intervention is the treatment of choice; however, fibrinolysis can be considered in those with ‘low-risk STEMI,’ defined by inferior STEMI with no right ventricular involvement or lateral AMI without hemodynamic compromise” [14]. If an unconfirmed COVID-19 patient develops non-ST-elevation MI (NSTEMI), it is better to do a diagnostic test before catheterization; the ACC further mentions that “conservative therapy may be sufficient in properly triaged patients with confirmed COVID-19. Hemodynamically unstable NSTEMI patients should be managed on the lines of those with STEMI” [14]. However, in many setups, the requirements for performing PCI in COVID-19 patients cannot be fulfilled. These requirements include wearing personal protective equipment, air-purifying respirators, and decontaminated and negative-pressure catheterization laboratory. Our patient had clinical symptoms, raised troponin, and hemodynamically stable but inferior STEMI with lateral wall involvement. We successfully treated our patient with streptokinase to decrease the chances of spreading infection in the hospital and due to unpreparedness to fulfill the COVID-19 protocol.

## Conclusions

Hypercoagulability and thromboembolic events in COVID-19 lead to pulmonary and cardiac complications. cTnI, cTnT, and BNP can be used to predict COVID-19 outcomes and deteriorating health. Those with higher cTnI levels have statistically significantly higher mortality as compared to others with normal levels. In many setups, the requirements for performing PCI in COVID-19 patients cannot be fulfilled. In such situations, streptokinase can be considered to curb the spread of infection in the hospital and when unprepared to fulfill the COVID-19 protocol.
